# Utility of serum uric acid levels in excluding pulmonary hypertension in severe chronic lung disease: insights from a tertiary care center

**DOI:** 10.1007/s10238-024-01488-9

**Published:** 2024-09-13

**Authors:** Shimon Izhakian, Alon Gorenshtein, Haya Engelstein, Lev Freidkin, Dror Rosengarten, Ofir Eldar, Mordechai R. Kramer

**Affiliations:** 1 Pulmonary Institute, Rabin Medical Center–Beilinson Hospital, 39 Jabotinski St., 4941492 Petach Tikva, Israel; 2 Faculty of Medicine, Tel Aviv University, 6997801 Tel Aviv, Israel

**Keywords:** Hyperuricemia, Uric acid, Pulmonary hypertension, Lung disease, WHO group 3

## Abstract

Hyperuricemia is a known predictor of World Health Organization (WHO) Group 1 pulmonary hypertension (PH) (pulmonary arterial hypertension), but its role in excluding PH secondary to chronic lung diseases (WHO Group 3) remains unclear. We retrospectively analyzed data from 323 patients with severe chronic pulmonary diseases who underwent evaluation for lung transplantation at a tertiary medical center between June 2017 and February 2023. We examined the association between hyperuricemia (serum uric acid > 6 mg/dL or > 0.357 mmol/L) and PH [mean pulmonary arterial pressure (MPAP) > 20 mmHg]. Compared to the normouricemia group (n = 211), hyperuricemic patients (n = 112) were more likely to be younger (*P* = 0.02), male (*P* < 0.001), and present with PH (*P* = 0.001) and severe PH (MPAP > 35 mmHg; *P* < 0.001). These patients also had a higher body mass index (*P* = 0.004), plasma N-terminal pro-B-type natriuretic peptide (*P* < 0.001), serum creatinine (*P* < 0.001), and C-reactive protein levels (*P* = 0.03). Significant associations with PH included higher body mass index (*P* = 0.005), uric acid levels (P < 0.001), total lung capacity (*P* = 0.02), and residual volume (*P* = 0.01); shorter 6-min walk test distance (*P* = 0.005); and lower forced expiratory volume in one second (*P* = 0.006) and diffusing capacity for carbon monoxide (*P* < 0.001). Multivariate analysis showed elevated uric acid levels remained significantly associated with PH (OR 1.29, 95% CI 1.05–1.58, *P* = 0.01). In conclusion, normal serum uric acid levels serve as a significant predictor for excluding pulmonary hypertension in patients with severe chronic lung diseases.

## Introduction

Chronic respiratory diseases are a major cause of morbidity and rank as the third leading cause of death worldwide [1]. Pulmonary hypertension (PH) is prevalent among patients with various forms of chronic lung disease and serves as an indicator of poor prognosis [2–4]. Right heart catheterization (RHC) is acknowledged as the gold standard for diagnosing and classifying PH [4], but it is an invasive procedure and is not routinely performed in patients with advanced chronic lung diseases [2–4]. While RHC remains the gold standard, echocardiography can estimate PH and is less invasive.

A variety of biomarkers, including serum uric acid, are recommended for the assessment of established PH [4]. Elevated serum levels of uric acid are commonly observed in patients with World Health Organization (WHO) group 1 PH (pulmonary arterial hypertension) and strongly correlate with disease severity [5–10]. However, information regarding the relationship of serum uric acid with PH associated with chronic lung diseases (WHO Group 3) is scarce. In the single available study comprising 212 patients with interstitial lung disease, the presence of hyperuricemia increased the probability of diagnosing PH [11].

We hypothesized that in a patient population with a broad spectrum of advanced chronic lung diseases, evaluation of serum uric acid levels could help clinicians predict or exclude PH when considering the need for RHC. The aim of this study was to assess the potential association of serum uric acid with PH among patients with severe chronic lung disease awaiting lung transplantation.

## Materials and methods

### Study population and design

The target study population comprised consecutive adult patients with various severe chronic lung diseases who were evaluated for eligibility for lung transplantation from June 2017 to February 2023 at Rabin Medical Center, the national center for lung transplantation in Israel. The transplant candidates were selected and examined according to the International Society for Heart and Lung Transplantation guidelines [12]. The evaluation included a detailed medical interview, physical and laboratory examination, six-minute walk test (6MWT), lung function tests (LFTs), computed tomography of the chest, and echocardiography. Only patients who had undergone RHC and had available serum uric acid measurements were included. For analysis of the association of hyperuricemia with PH and other relevant parameters, the patients were stratified into two groups based on serum levels of uric acid: normal (≤ 6 mg/dL) or elevated (> 6 mg/dL). The study was carried out in accordance with the Declaration of Helsinki and was approved by the institutional Ethics Committee (certification number: RMC-0373–24). Due to the retrospective nature of the study, informed consent was not required.

### Data collection

Baseline characteristics were collected retrospectively from the electronic medical records. The following demographic and clinical data were recorded: age, sex, lung disease diagnosis, body-mass index (BMI), and 6MWT distance. Laboratory data were obtained as follows: levels of serum creatinine, uric acid, C-reactive protein (CRP), and plasma N-terminal pro-B-type natriuretic peptide (NT-proBNP). The lung function test (LFT) parameters recorded included forced expiratory volume in one second (FEV1), forced vital capacity (FVC), total lung capacity (TLC)—measured by plethysmography in all cases—residual volume (RV), and diffusing lung capacity for carbon monoxide (DLCO). RHC data included cardiac output (CO), cardiac index (CI), pulmonary arterial wedge pressure (PAWP), mean pulmonary arterial pressure (MPAP), and pulmonary vascular resistance (PVR).

### Definitions

Hyperuricemia was defined as a serum uric acid level above the reference value (> 6 mg/dL) in our laboratory. PH was defined as a MPAP greater than 20 mmHg at rest. According to the 2022 ESC/ERS Guidelines for the Diagnosis and Treatment of Pulmonary Hypertension (4), we used PVR to differentiate between non-severe PH (PVR ≤ 5 Wood units [WU]) and severe PH (PVR > 5 WU). Pre-capillary PH was defined as MPAP > 20 mmHg, PAWP ≤ 15 mmHg, and PVR > 2 WU. Post-capillary PH was defined as MPAP > 20 mmHg and PAWP > 15 mmHg.

### Statistical analysis

The results were summarized as mean and standard deviation for quantitative data and as number (percentage) of cases for qualitative data. Chi-square test was used to compare categorical variables, and Student t test, to compare continuous variables. Statistical comparisons of the collected data were conducted between groups of patients with normouricemia and hyperuricemia. Correlation coefficient (r) was calculated to evaluate correlations of uric acid with BMI, creatinine, and LFT and RHC parameters. Pearson’s correlation coefficient (r) was calculated for normally distributed parameters, while Spearman’s rank correlation coefficient was used for correlations involving non-normally distributed data. *P* values ≤ 0.05 were considered significant.

Univariate analysis was conducted to identify variables significantly associated with PH (MPAP > 20 mmHg). Relevant variables found to be significant on univariate analysis were entered into a multivariate logistic regression model to identify those most significantly associated with PH. Additionally, we performed a univariate analysis specifically for patients with severe PH (PVR > 5 WU), excluding those with mild PH, and based on these findings, we generated a multivariate analysis. The statistical analysis was carried out using SAS software, version 9.2 (SAS Institute Inc., Cary, NC, USA).

## Results

### Baseline patient characteristics

Out of 458 patients with severe chronic lung disease under evaluation for lung transplantation during the study period, 135 were excluded from the analysis for the following reasons: 111 were deemed unfit for lung transplantation and did not undergo right heart catheterization (RHC), 18 were missing serum uric acid measurements, and 6 had WHO group 1 pulmonary hypertension (Primary PH). This left 323 patients eligible for analysis. Table 1 presents their baseline characteristics. The mean age was 62.2 ± 7.7 years, and 66.3% were male. The most common pulmonary disease diagnoses were interstitial lung disease (n = 176) and chronic obstructive pulmonary disease (COPD) (n = 121).

**Table 1:** Baseline characteristics of 323 lung transplant candidates with severe chronic lung disease, whole cohort and by serum uric acid level

Characteristic	Entire group (n = 323)	Normouricemia (n = 211)	Hyperuricemia (n = 112)	*P* value*
Age (yrs)	62.2 ± 7.7	62.9 ± 7.4	60.8 ± 8.0	0.02
Male sex	214 (66.3%)	124 (58.8%)	90 (80.4%)	< 0.001
*Comorbidity*				
Hypertension, n (%)	94 (29.1%)	59 (27.9%)	35 (31.2%)	0.2
Diabetes mellitus, n (%)	92 (28.4%)	60 (28.4%)	32 (28.5%)	0.4
Cerebrovascular disease, n (%)	12 (3.7%)	7 (3.3%)	5 (4.4%)	0.9
Ischemic heart disease, n (%)	51 (15.7)	30 (14.2%)	21 (18.7%)	0.3
Positive smoking history, n (%)	209 (64.7%)	129 (61.1%)	80 (71.4%)	0.07
BMI (kg/m^2^)	26.4 ± 5.4	25.8 ± 5.6	27.6 ± 4.8	0.004
*Chronic lung disease*				
Chronic interstitial lung disease	176 (54.5%)	113 (53.6%)	63 (56.3%)	0.64
COPD	121 (37.5%)	84 (39.8%)	37 (33.0%)	0.23
Silicosis	9 (2.7%)	6 (2.8%)	3 (2.6%)	0.30
*Laboratory data*				
Serum creatinine (normal 0.5–0.9 mg/dL)	1.32 ± 0.2	1.26 ± 0.2	1.44 ± 0.2	< 0.001
Serum uric acid (normal 2.4–6.0 mg/dL)	5.5 ± 1.9	4.4 ± 1.0	7.5 ± 1.4	< 0.001
Serum CRP (normal 0–0.5 mg/dL	0.84 ± 0.3	0.77 ± 0.2	0.96 ± 0.5	0.03
Plasma NT-proBNP (pg/mL)	358.1 ± 1155.7	158.2 ± 278.4	737.7 ± 1873.2	< 0.001
6MWT distance (meters)	271.8 ± 130.4	270.4 ± 126.7	274.4 ± 137.8	0.86
*Data of lung function test*				
FEV1 (% of predicted value)	42.3 ± 19.7	42.1 ± 20.2	42.6 ± 18.7	0.68
FVC (% of predicted value)	51.7 ± 16.9	51.8 ± 16.7	51.4 ± 17.3	0.51
TLC (% of predicted value)	81.9 ± 37.0	83.6 ± 38.4	78.7 ± 34.4	0.27
RV (% of predicted value)	138.6 ± 96.9	142.9 ± 98.7	130.9 ± 93.4	0.45
DLCO (% of predicted value)	32.9 ± 13.4	33.2 ± 13.4	32.4 ± 13.3	28
*Data of right heart catheterization*				
CO (1/min)	4.4 ± 1.3	4.2 ± 1.2	4.5 ± 1.4	0.11
CI (1/min/m^2^)	2.38 ± 0.6	2.4 ± 0.6	2.35 ± 0.6	0.61
PAWP (mmHg)	9.9 ± 6.3	8.9 ± 5.8	11.7 ± 6.7	< 0.001
MPAP (mmHg)	24.8 ± 10.2	22.7 ± 8.7	28.9 ± 11.5	< 0.001
PVR (WU)	3.7 ± 2.5	3.4 ± 1.9	4.2 ± 3.3	0.15
PH (MPAP > 20 mmHg), n(%)	223 (69.0%)	133 (63.0%)	90 (80.4%)	< 0.001

Data are presented as means ± standard deviations or numbers (percentages) of presented cases. *Difference between normouricemic and hyperuricemic groups. CRP: C-reactive protein; NT-proBNP: Bold entries in the table indicate a *P*-value of ≤ 0.05.

6MWT, 6-min walk test; BMI, body mass index; CI, cardiac index; CO, cardiac output; COPD, chronic obstructive pulmonary disease; CRP, C-reactive protein; DLCO, diffusing lung capacity for carbon monoxide; FEV1, forced expiratory volume in one second; FVC, forced vital capacity; MPAP, mean pulmonary artery pressure; NT-proBNP, N-terminal pro-B-type natriuretic peptide; PAWP, pulmonary arterial pressure; PH, pulmonary hypertension; PVR, pulmonary vascular resistance; RV, residual volume; TLC, total lung capacity; WU, Wood units

112 patients had no PH with a mean uric acid level of 5.0 ± 1.6 mg/dL. Among the 211 patients with PH (MPAP > 20 mmHg), the mean uric acid level was 5.7 ± 2.0 mg/dL. Of these, 155 patients had pre-capillary PH, with a mean uric acid level of 5.59 ± 2.1 mg/dL, while 56 patients had post-capillary PH, with a mean uric acid level of 6.1 ± 1.7 mg/dL. Among the 211 patients with PH, 155 had non-severe PH (PVR ≤ 5 Wood units [WU]), with a mean uric acid level of 5.6 ± 1.9 mg/dL, and 56 had severe PH (PVR > 5 WU), with a mean uric acid level of 6.0 ± 2.2 mg/dL.

### Groups comparison

Serum uric acid levels were within normal range in 211 patients and elevated in 112 patients (Table 1). Compared to patients with normouricemia, patients with hyperuricemia were more likely to be younger (*P* = 0.02) and male (*P* < 0.001). They also exhibited higher values of BMI (*P* = 0.004), plasma NT-proBNP (*P* < 0.001), serum creatinine (*P* < 0.001), and CRP (*P* = 0.03), as well as elevated PAWP (*P* < 0.001) and MPAP (*P* < 0.001). A higher percentage of patients in the hyperuricemia than the normouricemia group had PH (80.4% vs. 63.0%, *P* = 0.001) and severe PH (29.5% vs. 8.5%, *P* < 0.001).

### Correlation analysis

Table 2 shows the correlations between serum uric acid levels and the relevant baseline variables. Uric acid positively correlated with BMI (r = 0.32, *P* < 0.001), serum creatinine (r = 0.49, *P* < 0.001), CO (r = 0.19, *P* < 0.001), PAWP (r = 0.22, *P* < 0.001), MPAP (r = 0.29, *P* < 0.001), and PVR (r = 0.14, *P* = 0.01). An inverse correlation of uric acid was observed with RV (r = -0.10, *P* = 0.03).

**Table 2:** Correlations between serum levels of uric acid and other baseline parameters in lung transplant candidates with chronic lung disease

Parameter	Correlation coefficient (r)	*P*-value
BMI (kg/m^2^)	0.32	** < 0.001**
Serum creatinine (mg/dL)	0.49	** < 0.001**
6MWT distance (meters)	− 0.01	0.82
FEV1 (% of predicted value)	0.07	0.17
FVC (% of predicted value)	0.08	0.11
TLC (% of predicted value)	− 0.08	0.07
RV (% of predicted value)	− 0.10	**0.03**
DLCO (% of predicted value)	0.01	0.86
CO (L/min)	0.19	** < 0.001**
CI (L/min/m^2^)	− 0.02	0.67
PAWP (mmHg)	0.22	** < 0.001**
MPAP (mmHg)	0.29	** < 0.001**
PVR (WU)	0.14	**0.01**

^*^Bold entries in the table indicate a *P*-value of ≤ 0.05.

6MWT, 6-min walk test; BMI, body mass index; CI, cardiac index; CO, cardiac output; DLCO, diffusing lung capacity for carbon monoxide; FEV1, forced expiratory volume in one second; FVC, forced vital capacity; MPAP, mean pulmonary artery pressure; PAWP, pulmonary arterial wedge pressure; PH, pulmonary hypertension; PVR, pulmonary vascular resistance; RV, residual volume; TLC, total lung capacity; WU, Wood units

### Univariate analysis for prediction PH

Table 3 presents the results of the univariate analysis for predicting pulmonary hypertension (PH) in the entire cohort, as well as in ILD and COPD patients. In the overall cohort, the following variables were found to be significantly associated with PH: higher BMI (*P* = 0.005), serum uric acid (*P* < 0.001), shorter 6MWT distance (*P* = 0.005), FEV1 (*P* = 0.006), TLC (*P* = 0.02), RV (*P* = 0.01), DLCO (*P* < 0.001), and PAWP (*P* < 0.001).

**Table 3:** Variables evaluated for association with pulmonary hypertension (MPAP >20 mmHg) in lung transplant candidates: univariate analysis for overall cohort, interstitial lung disease (ILD), and chronic obstructive pulmonary disease (COPD)

Univariate analysis			
Variable	OR, 95% CI, *P*-value		
	Overall	ILD	COPD
Number of patients	323	176	121
Age (yrs)	0.97, 0.94–1.01, 0.13	0.98, 0.94–1.02, 0.5	0.97, 0.91–1.04, 0.5
Male sex	2.36, 0.51–1.36, 0.48	1.24, 0.51–2.63, 0.6	1.24, 0.51–3.03, 0.6
BMI (kg/m^2^)	1.06, 1.02–1.11, **0.005**	1.07, 1.01–1.14, **0.02**	1.11, 1.01–1.22, **0.02**
Serum creatinine (mg/dL)	1.07, 0.52–2.19, 0.84	1.5, 0.54–4.17, 0.4	0.97, 0.91–1.04, 0.5
Serum uric acid (mg/dL)	1.29, 1.11–1.49, < **0.001**	1.37, 1.10–1.70, **0.004**	1.13, 0.9–1.43, 0.28
Serum CRP (mg/dL)	1.05, 0.92–1.20, 0.43	1.04, 0.84–1.28, 0.6	1.25, 0.85–1.84, 0.2
Plasma NT-proBNP (pg/mL)	1.00, 1.00–1.00, 0.15	1.01, 1.00–1.01, **0.03**	1.00, 0.99–1.00, 0.2
6MWT distance (meters)	0.99, 0.99–1.00, **0.005**	0.99, 0.99–0.99, **0.004**	1.00, 0.99–1.00, 0.8
FEV1 (% of predicted value)	0.98, 0.97–0.99, **0.006**	0.99, 0.97–1.01, 0.2	0.98, 0.95–1.01, 0.2
FVC (% of predicted value)	1.00, 0.99–1.01, 0.97	1.00, 0.96–1.06, 0.5	0.99, 0.96–1.03, 0.3
TLC (% of predicted value)	1.01, 1.00–1.01, **0.02**	1.02, 1.01–1.04, **0.04**	0.98, 0.96–1.00, 0.08
RV (% of predicted value)	1.00, 1.00–1.01, **0.01**	1.01, 1.00–1.02, **0.03**	0.99, 0.99–1.00, 0.3
DLCO (% of predicted value)	0.96, 0.95–0.98, < **0.001**	0.95, 0.93–0.98, **0.001**	0.96, 0.93–1.00, 0.06
CO (L/min)	1.09, 0.90–1.33, 0.36	1.04, 0.81–1.33, 0.72	1.24, 0.82–1.89, 0.2
CI (L/min/m^2^)	0.91, 0.60–1.39, 0.67	0.87, 0.51–1.49, 0.6	0.75, 0.31–1.80, 0.5
PAWP (mmHg)	1.36, 1.27–1.47, < **0.001**	1.36, 1.23–1.51, < **0.001**	1.36, 1.19–1.56, < **0.001**
PVR (WU)	1.47, 0.99–1.01, 0.36	1.84, 1.40–2.41, < **0.001**	1.19, 0.91–1.57, 0.19
*Multivariate analysis*			
*Variable*	*OR, 95% CI, P-value*		
Age (yrs)	0.98, 0.94–1.02, 0.33	1.00, 0.94–1.06, 0.9	–
Male sex	1.48, 1.00–3.07, 0.28	0.27, 0.08–0.83, **0.02**	–
BMI (kg/m^2^)	1.08, 1.02–1.15, **0.007**	1.04, 0.95–1.13, 0.3	–
Serum creatinine (mg/dL)	0.34, 0.08–1.34, 0.12	0.16, 0.01–2.09, 0.1	–
Serum uric acid (mg/dL)	1.3, 1.09–1.54, **0.01**	1.44, 1.05–1.96, **0.02**	–
Plasma NT-proBNP (pg/mL)	1.00, 1.00–1.00, 0.39	1.00, 0.99–1.00, 0.2	–
6MWT distance (meters)	0.99, 0.99–1.00, 0.24	0.99, 0.99–1.00, **0.05**	–
DLCO (% of predicted value)	1.04, 1.06–1.09, **0.007**	0.96, 0.93–1.00, 0.08	–

^*^Bold entries in the table indicate a *P*-value of ≤ 0.05.

ILD, interstitial lung disease; COPD, chronic obstructive pulmonary disease; 6MWT, 6-min walk test; BMI, body mass index; CI, cardiac index; CO, cardiac output; DLCO, diffusing lung capacity for carbon monoxide; FEV1, forced expiratory volume in one second; FVC, forced vital capacity; MPAP, mean pulmonary artery pressure; NT-proBNP, N-terminal pro-B-type natriuretic peptide; PAWP, pulmonary arterial wedge pressure; PH, pulmonary hypertension; PVR, pulmonary vascular resistance; RV, residual volume; TLC, total lung capacity; WU, Wood units

Among ILD patients, the variables significantly associated with PH were BMI (*P* = 0.02), serum uric acid (*P* = 0.004), NT-proBNP (*P* = 0.03), shorter 6MWT distance (*P* = 0.004), TLC (*P* = 0.04), RV (*P* = 0.03), DLCO (*P* = 0.001), PAWP (*P* < 0.001), and PVR (*P* < 0.001) (Table 3).

For COPD patients, the variables significantly associated with PH were BMI (*P* = 0.02) and PAWP (P < 0.001) (Table 3).

### Multivariate analysis for prediction PH

In the multivariate logistic regression analysis conducted on the entire patient cohort, considering covariates such as age, sex, BMI, serum creatinine, uric acid, plasma NT-proBNP, 6MWT distance, and DLCO, the variables most significantly associated with PH were elevated uric acid (OR 1.30, 95% CI 1.09–1.54, *P* = 0.01), higher BMI (OR 1.08, 95% CI 1.02–1.15, *P* = 0.007), and lower DLCO (OR 1.04, 95% CI 1.06–1.09, *P* = 0.007) (Table 3).

In the same multivariate logistic regression analysis, but focusing only on ILD patients, the significant parameters were male sex (OR 0.27, 95% CI 0.08–0.83, *P* = 0.02), serum uric acid (OR 1.44, 95% CI 1.05–1.96, *P* = 0.02), and 6MWT distance (OR 0.99, 95% CI 0.99–1.00, *P* = 0.05) (Table 3).

We did not perform a multivariate analysis for COPD patients because serum uric acid levels were not significant in the univariate analysis.

### Univariate and Multivariate Analysis for Severe PH

Table 4 presents the univariate and multivariate analyses evaluating the association of uric acid with severe PH (PVR > 5 WU) in lung transplant candidates. Serum uric acid level, when considered as a continuous variable, was not significant; therefore, we evaluated it as a dichotomous variable using a cutoff of > 6 mg/dL, based on the reference level.

**Table 4:** Variables evaluated for association with sever PH (PVR>5 WU) in lung transplant candidates with severe chronic pulmonary disease univariate and multivariate analysis

Univariate analysis			
Variable	OR	95% CI	*P*-value
Age (yrs)	0.99	0.96–1.03	0.9
Male sex	1.66	0.93–2.96	0.08
BMI (kg/m^2^)	0.99	0.94–1.05	0.9
Serum creatinine (mg/dL)	1.02	0.43–2.37	0.9
Serum uric acid (mg/dL)	1.13	0.96–1.32	0.1
Serum uric acid > 6 mg/dL	1.93	1.07–3.45	**0.02**
Serum CRP (mg/dL)	1.12	0.98–1.27	0.08
Plasma NT-proBNP (pg/mL)	1.00	1.00–1.01	0.05
6MWT distance (meters)	0.99	0.99–1.00	0.01
FEV1 (% of predicted value)	1.01	0.99–1.02	0.1
FVC (% of predicted value)	1.01	0.99–1.03	0.1
TLC (% of predicted value)	1.01	0.99–1.01	0.8
RV (% of predicted value)	0.99	0.99–1.00	0.6
DLCO (% of predicted value)	0.94	0.92–.97	**0.001**
CO (L/min)	0.35	0.24–0.51	** < 0.001**
CI (L/min/m2)	0.15	0.07–0.31	** < 0.001**
PAWP (mmHg)	0.99	0.94–1.03	0.7
*Multivariate analysis*			
*Variable*	*OR*	*95% CI*	*P-value*
Age (yrs)	1.03	0.98–1.09	0.1
Male sex	1.41	0.59–3.37	0.4
BMI (kg/m^2^)	0.96	0.90–1.04	0.3
Serum creatinine (mg/dL)	0.2	0.05–1.39	0.1
Serum uric acid > 6 mg/dL	1.44	1.13–1.82	**0.002**
Plasma NT-proBNP (pg/mL)	1	1.00–1.00	0.1
6MWT distance (meters)	0.99	0.99–1.00	0.2
DLCO (% of predicted value)	0.96	0.93–0.99	**0.03**

^*^Bold entries in the table indicate a P-value of ≤ 0.05.

PH, pulmonary hypertension, WU, Wood units; 6MWT, 6-min walk test; BMI, body mass index; CI, cardiac index; CO, cardiac output; DLCO, diffusing lung capacity for carbon monoxide; FEV1, forced expiratory volume in one second; FVC, forced vital capacity; MPAP, mean pulmonary artery pressure; NT-proBNP, N-terminal pro-B-type natriuretic peptide; PAWP, pulmonary arterial wedge pressure; PH, pulmonary hypertension; PVR, pulmonary vascular resistance; RV, residual volume; TLC, total lung capacity

In the univariate analysis for predicting severe pulmonary hypertension, the following variables were significant: uric acid level > 6 mg/dL (*P* = 0.02), DLCO (*P* < 0.001), cardiac output (CO) (*P* < 0.001), and cardiac index (CI) (*P* < 0.001) (Table 4).

In the multivariate logistic regression analysis conducted on the entire patient cohort, which considered covariates such as age, sex, BMI, serum creatinine, uric acid, plasma NT-proBNP, 6MWT distance, and DLCO, the variables most significantly associated with PH were elevated uric acid > 6 mg/dL (OR 1.44, 95% CI 1.13–1.82, P = 0.002) and DLCO (OR 0.96, 95% CI 0.93–0.99, P = 0.03) (Table 4).

## Discussion

We evaluated the ability of serum uric acid level to serve as a predictor of PH in 323 patients with severe chronic pulmonary disease, all candidates for lung transplantation. A significant and novel aspect of our investigation is the demonstration that elevated levels of uric acid were strongly associated with the presence of WHO group 3 PH.

Our findings align with the study of Andersen et al. [11] wherein an association was found between hyperuricemia and the presence of PH in 212 patients with interstitial lung disease. However, there are notable differences between the present and earlier study in design, population characteristics, and results. Firstly, our investigation comprised a larger sample and included patients with a broad spectrum of severe chronic pulmonary diseases, reflecting a real-world population of lung transplant candidates. Secondly, in our study, every patient underwent RHC, ensuring a comprehensive evaluation, whereas in the previous report, RHC was conducted in only 17 patients, potentially leading to measurement imprecision due to reliance on echocardiography (ECHO) for PH diagnosis. Thirdly, we confirmed the robust association of increased uric acid levels with PH by multivariate analysis, accounting for crucial confounders such as age, sex, BMI, serum creatinine level, plasma NT-proBNP level, 6MWT distance, and DLCO.

We specifically assessed the association of serum uric acid with severe PH (MPAP > 35 mmHg) and determined an optimal cut-off of 5.7 mg/dL, with an AUC of 0.72. Seyyedi et al. [7] reported an identical optimal cut-off for serum uric acid levels in predicting severe PH in a cohort of 110 patients with established PH of various etiologies. RHC is not routinely performed in patients with severe chronic lung diseases, but rather selectively undertaken for those with high clinical and echocardiographic suspicion of severe PH. Our findings suggest that in this patient population, measuring serum uric acid levels can be a useful tool for ruling out severe PH. Furthermore, we propose that assessing uric acid levels in lung transplant candidates may aid physicians in decision-making regarding the need for RHC for diagnosing severe PH, given the known therapeutic benefits of bilateral lung transplantation for severe PH.

The pathophysiological mechanisms underlying the relationship between hyperuricemia and PH associated with chronic lung diseases (WHO group 3) are not fully understood. The main proposed cause of elevated serum uric acid in patients with severe chronic pulmonary disease is tissue hypoxia [5, 10]. Specifically, uric acid is the final product of xanthine-oxidase-generated purine metabolism, and tissue hypoxia increases this process by inducing a decrease in adenosine triphosphate synthesis [5, 10]. An additional contributing factor of hyperuricemia in patients with chronic lung disease may be concomitant metabolic syndrome and renal dysfunction [5, 13]. In our patients, hyperuricemia was indeed associated with higher values of BMI and serum creatinine.

Hyperuricemia may itself play a role in the development of PH in chronic lung diseases. Firstly, hyperuricemia induces endothelial dysfunction by decreasing the production of nitric oxide [5, 10, 14]. As a result, the release of various vasoactive substances and cytokines from endothelial cells is increased, leading to pulmonary vasoconstriction and the development of PH [5, 10, 15]. Secondly, hyperuricemia promotes smooth muscle cell proliferation and inflammation by increasing the production of uric acid in pulmonary artery smooth muscle cells. This increase is a result of enhanced activity of xanthine oxidase and decreased expression of the voltage-driven urate transporter 1 [5, 10, 16, 17]. Third, elevated concentrations of serum uric acid are associated with activation of the renin–angiotensin–aldosterone system, which may contribute to impaired vascular remodeling and aggravated pulmonary vasoconstriction, resulting in increased pressure in the pulmonary artery [5, 10, 18].

Additionally, a recent study has shown that serum uric acid levels are elevated in patients with silicosis and pulmonary heart disease compared to those with silicosis alone. This finding suggests that serum uric acid may serve as a marker for the severity of pulmonary heart disease and highlights its role in the disease’s progression [19]. Finally, the development of PH in patients with chronic lung diseases could aggravate tissue hypoxia and oxidative stress, leading to hyperuricemia, which may, in turn, may further worsen PH [3, 10, 20].

An additional interesting observation in our study is the association of hyperuricemia with male sex, as well as higher values of serum CRP and plasma NT-proBNP. Our findings for male sex are in line with data from two studies on patients with COPD [21, 22]. The association of hyperuricemia with higher CRP concentrations likely reflects its proinflammatory effect [5, 17] and has been reported in a large cohort of community-dwelling patients [23].

To our knowledge, among patients with chronic lung diseases, the association of hyperuricemia with increased NT-proBNP levels has not been reported. However, a positive correlation between concentrations of uric acid and NT-proBNP has been observed in patients with left heart failure [24, 25]. The pathophysiological mechanism responsible for the association of hyperuricemia with NT-proBNP elevation in chronic lung disease is unclear. We suggest that in patients with severe pulmonary disorders, hyperuricemia promotes the development of WHO group 3 PH, while PH and right ventricular dysfunction cause an increase in NT-proBNP.

We found that uric acid levels were slightly higher in patients with PH and increased PAWP compared to those with reduced PAWP. We acknowledge the possibility that some of the observed increase in NT-proBNP levels and uric acid could be related to left heart disease, particularly in cases where PH is not extremely elevated. Elevated NT-proBNP levels might reflect high pulmonary arterial wedge pressure rather than right ventricular strain alone. However, it is crucial to emphasize that our study specifically excluded patients with significant left heart disease symptoms, such as pleural effusion, reduced ejection fraction, and leg edema. These patients are generally not considered for lung transplantation. Therefore, while left heart disease could potentially influence NT-proBNP levels and uric acid, our findings predominantly reflect the association of hyperuricemia with PH in the context of severe chronic lung diseases, supporting the relevance of our results in this specific patient population.

The present study has several limitations. Firstly, since the investigation was conducted at a single medical center, its generalizability to other medical centers is uncertain. Secondly, data collection was limited to a single timepoint during the evaluation for lung transplantation, which may introduce misclassification due to time-dependent changes in the variables obtained. Thirdly, specific echocardiographic measurements were not included in the data collection section of the manuscript. This is because the echocardiograms were performed at different locations with varying quality and were interpreted subjectively by a range of cardiologists. Consequently, the variability in imaging quality and interpretation limited the inclusion of these data in our analysis. Finaly, while this study offers a broad overview of various respiratory diseases and both precapillary and postcapillary pulmonary hypertension, it is important to acknowledge that this approach may oversimplify the complexity of these conditions. Future research with a more focused approach, targeting specific disease mechanisms and subtypes, is needed to provide a deeper understanding and more precise insights.

## Conclusion

Among patients with a broad spectrum of severe chronic pulmonary diseases who are candidates for lung transplantation, normal serum uric acid levels are a significant predictor for excluding the presence of pulmonary hypertension. Assessing serum uric acid levels in this population could assist physicians in the decision-making process regarding the necessity of right heart catheterization (RHC).

## Data Availability

No datasets were generated or analysed during the current study.
